# Numerical test concerning bone mass apposition under electrical and mechanical stimulus

**DOI:** 10.1186/1742-4682-9-14

**Published:** 2012-05-11

**Authors:** Diego A Garzón-Alvarado, Angélica M Ramírez-Martínez, Carmen Alicia Cardozo de Martínez

**Affiliations:** 1 Research Group on Numerical Methods for Engineering (GNUM), Universidad Nacional de Colombia, Bogota, Colombia; 2 Central University Bioengineering Group, Universidad Central de Colombia, Bogota, Colombia; 3 Biomimetics Laboratory. Biotechnonology Institute, Universidad Nacional de Colombia, Bogota, Colombia

## Abstract

This article proposes a model of bone remodeling that encompasses mechanical and electrical stimuli. The remodeling formulation proposed by Weinans and collaborators was used as the basis of this research, with a literature review allowing a constitutive model evaluating the permittivity of bone tissue to be developed. This allowed the mass distribution that depends on mechanical and electrical stimuli to be obtained. The remaining constants were established through numerical experimentation. The results demonstrate that mass distribution is altered under electrical stimulation, generally resulting in a greater deposition of mass. In addition, the frequency of application of an electric field can affect the distribution of mass; at a lower frequency there is more mass in the domain. These numerical experiments open up discussion concerning the importance of the electric field in the remodeling process and propose the quantification of their effects.

## Background

Bones provide mechanical stability to the human body and are a source of minerals for metabolism [1]. Bones have been studied extensively from the mechanical and mineral standpoint, and in terms of functionality [1,2]. From a mechanical point of view, bones can be adapted to loads on trajectories of stress through mineral apposition, which is due to the action of osteoblasts [1-4]. Furthermore, they reabsorb minerals when the mechanical stimulus is sufficiently low, as it is unnecessary to maintain structure [2]. Reabsorption is directed by osteoclasts. Osteoblasts and osteoclasts are the primary cells involved during bone remodeling that are stimulated by the action of mechanical strain sensors, for example, osteocytes [2]. These three cell types play an important role during the processes of replacement, maintenance and modeling of bones [1].

Following the work of Meyer during the nineteenth century, Wolff [5] proposed a theory of trabecular bone architecture, which assumes that trajectories of high mechanical stress form the trabecular bone. In 1987, Frost [6-8] suggested an adaptive mechanism of bone mass as a function of mechanical stress. Consequently, several bone remodeling algorithms have been developed including those proposed by Frost [8], Pauwels [9], Kummer [10], Cowin [11-13] and Hegedus [14], which predict the formation of bone structure from internal mechanical loads studied in terms of stress and strain.

From mechanical models of bone remodeling, sophisticated studies have been carried out concerning the processes of apposition and reabsorption during bone turnover, and particularly concerning the distribution of mass in the femur [15,16], hip replacement [17,18], and dental implants [19]. Generally, these studies have been phenomenological. Therefore, researchers have made significant efforts to include mathematical models and the role of cell biology and biochemistry in the remodeling process, resulting in research that begins at the microscopic level, concerning the effects of basic cellular remodeling units (BMU, Basic multicellular units) during tissue replacement [20,21]. From the perspective of BMU, important work was initiated at the biochemical and mechanical level concerning the effects of cracks [22], cell cycles throughout adult life [23], active molecules within each cell [24] and the spatial distribution of each BMU [25]. With these important advances in the understanding of bone remodeling, researchers in the field increasingly turned to the study of other biophysical stimuli that can affect this process. Most models have not taken account of the physical-chemical phenomena of tissue mechano-transduction. For this reason, new investigations that allow the piezoelectric and electrokinetic behavior of the bone to be studied were undertaken [26].

A clinical study demonstrated that a local electromagnetic field accelerates the healing process after bone fracture [26]. Therefore, an article by Demiray and Dost [27] began new research concerning the effect of the electromagnetic field on interior injury to bone. In another article, Ramtani [26] presented a mathematical model relating to the benefit of the electric field in the reparair and maintenance of the solid matrix of bone. Furthermore, there are studies concerning the electrical behavior of bone tissue during the production of electric fields, and external electrical flow. Fukada and Yasuda [28] demonstrated that bone exhibits piezoelectric behavior, i.e. mechanical stress creates electric polarization (the indirect effect) and an external electric field causes strain (the converter effect). In addition, the properties of bones that produce piezoelectric potentials have been determined [29-33]. These data led to the development of mathematical models that include the effect of electromagnetic fields during the repair [34,35] and remodeling [36] of bone. For example, Qu and Yu [34] developed a mathematical model (no spatial dimension) of the remodeling process and healing under the effect of mechanical loads and the use of electric charges. In this model, the higher the voltage applied to a bone after fracture, the lower the percentage of bone damage and micro damage in the few days after the stimulus. Similarly, during osteoporosis an electric field increases bone density over time. Huang et al. [37] established a hypothesis concerning the biological and biochemical pathways that activate cells, particularly osteocytes, during the imposition of an electric field. Furthermore, Qu and Yu [38] proposed a mathematical model that included mechanical loads and electromagnetic effects during the process of bone remodeling.

To date, there have been no phenomenological models concerning bone remodeling that have been tested and compared with purely mechanical models. Therefore, this article proposes a new electro-mechanical model relating to bone remodeling. To test its performance, various numerical experiments were carried out and compared with previous mechanical models. The electric model constants were obtained from relevant literature and numerical experimentation. From these assumptions it was concluded that the electric field can affect the distribution of mass, which originates from the remodeling process, under mechanical effects only. Using the remodeling model of Nackenhorst [39] as a starting point, it was demonstrated that the electric field can increase bone density and accelerate the process of apposition. Therefore, the model proposed herein can be used as a basis for further work concerning electrical effects in the maintenance of bone.

## Methods

### The electro-mechanical model

The electro-mechanical model of bone remodeling that involves mechanical and electrical stimuli can be written, hypothetically, as follows (1):

(1) $$\frac{d\rho }{dt}=g_{\mathrm{mech}}\left(\rho ,W_{\mathrm{mec}}\left(\rho \right)\right)+g_{\mathrm{elect}}\left(\rho ,W_{\mathrm{elect}}\left(\varepsilon (\rho ,f)\right)\right)$$

Where $g_{\mathrm{mech}}\left(\rho ,W_{\mathrm{mec}}\left(\rho \right)\right)$ is the well known mechanical stimulus described by Weinans [39], which depends on tissue density ( ρ(x,y,z,t)), and the work carried out by mechanical stress ( $W_{\mathrm{mec}}(\rho )$) and $g_{\mathrm{elect}}\left(\rho ,W_{\mathrm{elect}}\left(\varepsilon (\rho ,f)\right)\right)$ is the electrical stimulus that depends on density, frequency and the work carried out by the electric field ( $W_{\mathrm{elect}}\left(\varepsilon (\rho )\right)$). During this first approach, we consider that the two stimuli are added to determine the bone remodeling process. However, we will develop each of the terms that determine the electro-mechanical model throughout this article.

### The mechanical model

Following the remodeling mechanical model described in [4,39], the density variation over time depends on the mechanical stimulus that exists at every spatial point of the bone, which can be written as in [39] (2):

(2) $$g_{\mathrm{mech}}\left(\rho ,W_{\mathrm{mec}}\left(\rho \right)\right)=k_{1}\left(\frac{W(\rho )}{W_{ref_{m}}}-1\right)$$

Where ρis the bone density at each point in space ( ρ(x,y,z,t)), W(ρ)is the energy-strain per unit of volume due to mechanical stress, $k_{1}$ is a constant and $W_{\mathrm{ref}}{}_{m}$ is the strain-energy (per unit of volume) of reference that sets the threshold for which they will perform deposition ( $W(\rho )/W_{ref_{m}}>1$) or absorption ( $W(\rho )/W_{ref_{m}}<1$) of the tissue [39], in the presence of mechanical stress. It should be noted that the energy-strain depends on the density and is given by (3):

(3) $$W(\rho )=\frac{1}{2\rho }\varepsilon^{T}C(\rho )\varepsilon$$

Where ε is the strain, in Voigt notation, of the strain tensor given in (4):

(4) $$\varepsilon ={\left[\begin{array}{ccc} \varepsilon_{11} & \varepsilon_{22} & \gamma_{12} \end{array}\right]}^{T}$$

That is a function of the displacements given by (5):

(5) $$u={\left[\begin{array}{cc} u_{1} & u_{2} \end{array}\right]}^{T}$$

(6) $$\varepsilon =B_{1}u=\left[\begin{array}{cc} \frac{\partial }{\partial x} & 0\\ 0 & \frac{\partial }{\partial y}\\ \frac{\partial }{\partial y} & \frac{\partial }{\partial x} \end{array}\right]\left[\begin{array}{c} u_{1}\\ u_{2} \end{array}\right]$$

In addition, C(ρ) is the matrix of linear elasticity. The matrix C(ρ) contains the Poisson module, which is usually considered constant, and Young's modulus that depends on the density by expression [4] (7):

(7) $$E(\rho )=A\rho^{n}$$

where A is a constant and n establishes a relationship of power density that has been uncovered through experimentation [39].

By manipulating equation (7) we can obtain a dimensionless form of the density. This is easier to use with the aim of determining the modulus of elasticity. Multiplying the right side of (7) by $\left(\rho_{0}^{n}/\rho_{0}^{n}\right)$ produces (8):

(8) $$E(\rho )=A\rho_{0}^{n}{\left(\frac{\rho }{\rho_{0}}\right)}^{n}=E_{0}{\left(\frac{\rho }{\rho_{0}}\right)}^{n}=E_{0}\lambda^{n}$$

Where $E_{0}=A\rho_{0}^{n}$ and $\lambda =\rho /\rho_{0}$ are the elasticity modulus and the dimensionless density ratio, respectively. Therefore, the linear elasticity matrix can be expressed as (9):

(9) $$C(\rho )=\lambda^{n}C_{0}$$

Where $C_{0}$ is the matrix of linear elasticity with constant coefficients, which depends on $E_{0}$ and ν only, and is given in the case of plane stress by:

(10) $$C_{0}=\frac{E_{0}}{1-\nu^{2}}\left[\begin{array}{ccc} 1 & \nu & 0\\ \nu & 1 & 0\\ 0 & 0 & \frac{1}{2}\left(1-\nu \right) \end{array}\right]$$

Thus, the strain energy per unit of volume (3) can be expressed as (11):

(11) $$W(\rho )=\frac{1}{2\rho }\lambda^{n}\varepsilon^{T}C_{0}\varepsilon =\frac{\lambda^{n}}{\rho }\left(\frac{\varepsilon^{T}C_{0}\varepsilon }{2}\right)=\frac{\lambda^{n}}{\rho }{\bar{U}}_{\mathrm{mec}}$$

Where ${\bar{U}}_{\mathrm{mec}}$ is the strain energy at each instant of time, which is calculated with the initial constant of the remodeling [39] problem only. Replacing these equations in (2), and with some algebraic manipulation, we produce (12):

(12) $$g_{\mathrm{mech}}\left(\rho ,W_{\mathrm{mec}}\left(\rho \right)\right)=k_{1}\left(\frac{\rho_{0}}{\rho_{0}}\frac{\lambda^{n}{\bar{U}}_{\mathrm{mec}}}{\rho W_{ref_{m}}}-1\right)=k_{1}\left(\left(\frac{\rho_{0}}{\rho }\right)\frac{\lambda^{n}{\bar{U}}_{\mathrm{mec}}}{\rho_{0}W_{ref_{m}}}-1\right)=k_{1}\left(\lambda^{n-1}\frac{{\bar{U}}_{\mathrm{mec}}}{\rho_{0}W_{ref_{m}}}-1\right)$$

where we can define $U_{ref_{m}}=\rho_{0}W_{ref_{m}}$. Therefore, we produce the following equation for the density ratio (13):

(13) $$g_{\mathrm{mech}}=k_{1}\left(\lambda^{n-1}\frac{{\bar{U}}_{\mathrm{mec}}}{U_{ref_{m}}}-1\right)$$

The momentum equation that establishes the internal stresses of a body is given by [40] (14):

(14) ∇·σ+b=0

Where the stress is given by (15):

(15) $$\sigma =C(\rho )\varepsilon =\lambda^{n}C_{0}\varepsilon$$

### Electrical model

This article proposes the inclusion of a hypothetical electrical term that can determine, in part, the process of bone remodeling. Therefore, the contribution of this stimulus can be written as (16):

(16) $$g_{\mathrm{elect}}\left(\rho ,W_{\mathrm{elect}}\left(\varepsilon (\rho ,f)\right)\right)=k_{2}\frac{W_{\mathrm{elect}}(\varepsilon (\rho ,f))}{W_{ref_{e}}}$$

Where ∈(ρ,f) is the electrical permittivity of bone tissue and depends on the density, ( ρ) where frequency ( f), $W_{\mathrm{elect}}(\in (\rho ,f))$ is the electrical energy per unit of volume, $k_{2}$ is a constant and $W_{\mathrm{ref}}{}_{e}$ is the electric energy (per unit volume) of reference.

It should be noted that the term electric energy depends on permittivity, which in turn depends on the density and frequency. This energy term is given by (17):

(17) $$W_{\mathrm{elect}}(\in (\rho ,f))=\frac{1}{2\rho }\in (\rho ,f){\left(E(x,y,z,t)\right)}^{2}$$

Where E(x,y,z,t) is the electric intensity (electric field). However, ε(ρ,f), electrical permittivity, depends on density and frequency, and is given by (18):

(18) $$\in (\rho ,f)=\in_{0}\in_{r}(\rho ,f)$$

Where $\in_{0}$ is the permittivity in the free space and $\in_{r}(\rho ,f)$ is the relative permittivity, which in turn is given by (19):

(19) $$\in_{r}(\rho ,f)=B\rho^{m}+\delta (f)$$

where B is a constant and m establishes a relationship of power density that can be proved through experimentation, and will be developed in the following sections. Furthermore, δ(f) is a function of frequency at which the electric field is applied.

As with the mechanical case, we can manipulate equation (19) so that it is expressed in terms of relative density, multiplying the first term by to produce $\left(\rho_{0}^{n}/\rho_{0}^{n}\right)$ (20):

(20) $$\in_{r}(\rho ,f)=B\rho_{0}^{m}\lambda^{m}+\delta (f)=\in_{\rho }\lambda^{m}+\delta (f)$$

where $\in_{\rho }$ is a constant for the power model of relative permittivity. Therefore, substituting (20) in (17), we obtain (21):

(21) $$\begin{array}{l} W_{\mathrm{elect}}(\in (\rho ,f))=\frac{1}{2\rho }\frac{\rho_{0}}{\rho_{0}}\in_{0}(\in_{\rho }\lambda^{m}+\delta (f)){\left(E(x,y,z,t)\right)}^{2}\\ =\frac{1}{2\rho_{0}\lambda }\in_{0}(\in_{\rho }\lambda^{m}+\delta (f)){\left(E(x,y,z,t)\right)}^{2}=\frac{1}{\rho_{0}\lambda }(\in_{\rho }\lambda^{m}+\delta (f)){\bar{U}}_{\mathrm{elect}} \end{array}$$

where ${\bar{U}}_{\mathrm{elect}}=\frac{1}{2}\in_{0}{\left(E(x,y,z,t)\right)}^{2}$. Substituting equation (21) in (16) produces (22):

(22) $$g_{\mathrm{elect}}\left(\rho ,W_{\mathrm{elect}}\left(\in (\rho )\right)\right)=k_{2}\frac{\frac{1}{\rho_{0}\lambda }(\in_{\rho }\lambda^{m}+\delta (f)){\bar{U}}_{\mathrm{elect}}}{W_{ref_{e}}}=k_{2}\frac{\left(\in_{\rho }\lambda^{m}+\delta (f)\right)}{\lambda }\frac{{\bar{U}}_{\mathrm{elect}}}{U_{ref_{e}}}$$

We have chosen $U_{ref_{e}}=\rho_{0}W_{ref_{e}}$ as the reference value of electric energy.

However, Gauss's law for an electric field, with no internal loads, is given by (23):

(23) $$\nabla \cdot D=\rho_{e}$$

where D=∈E and $\rho_{e}$ is the density of the electric energy. In turn, the electric field can be expressed in terms of a quantity called electric potential or voltage, given by (24):

(24) E=−∇ϕ

where ϕ is the electric potential.In summary, equation (1) can be written as (25):

(25) $$\frac{d\rho }{dt}=k_{1}\left(\lambda^{n-1}\frac{{\bar{U}}_{\mathrm{mec}}}{U_{ref_{m}}}-1\right)+k_{2}\frac{\left(\in_{\rho }\lambda^{m}+\delta (f)\right)}{\lambda }\frac{{\bar{U}}_{\mathrm{elect}}}{U_{ref_{e}}}$$

Again, using the dimensionless form, we have (26):

(26) $$\frac{d\lambda }{dt}=\frac{k_{1}}{\rho_{0}}\left(\lambda^{n-1}\frac{{\bar{U}}_{\mathrm{mec}}}{U_{ref_{m}}}-1\right)+\frac{k_{2}}{\rho_{0}}\frac{\left(\in_{\rho }\lambda^{m}+\delta (f)\right)}{\lambda }\frac{{\bar{U}}_{\mathrm{elect}}}{U_{ref_{e}}}$$

(27) $$\frac{d\lambda }{dt}=k_{\mathrm{mec}}\left(\lambda^{n-1}\frac{{\bar{U}}_{\mathrm{mec}}}{U_{ref_{m}}}-1\right)+k_{\mathrm{elect}}\frac{\left(\in_{\rho }\lambda^{m}+\delta (f)\right)}{\lambda }\frac{{\bar{U}}_{\mathrm{elect}}}{U_{ref_{e}}}$$

where $k_{\mathrm{mec}}=k_{1}/\rho_{0}$and $k_{\mathrm{elect}}=k_{2}/\rho_{0}$ are mechanical and electrical constants that define the conversion rate of bone remodeling, dependent on the mechanical stress and the electrical potential, respectively.

### Solution by the finite element method

To solve equation (14) we take its differential form to its weak [40] form, to obtain (27):

(28) $$\int_{\Omega }\delta \varepsilon \lambda^{n}C_{0}\varepsilon d\Omega -\int_{\Omega }\delta u\cdot bd\Omega -\int_{\Gamma }\delta u\cdot td\Gamma =0$$

**Figure 1:**
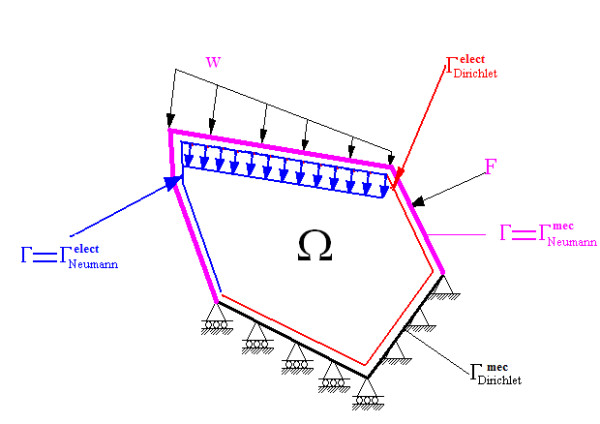
**Domain and boundary conditions.** The acronym on the index indicates whether it is a condition for the electric (elect) or mechanical (mec) case. Note that there are two types of boundaries (not necessarily equal) for each equation, the electric field and displacement (for the mechanical stress).

Where δε and δu are weighting functions, and t=σ·n is the traction on the boundary Γ that serves as a frontier to the domain Ω (see Figure 1).

To calculate the approximate solution by finite element discretization, the displacement field approximates through [40] (28):

(29) $$u(x,t)=N^{T}(x)a(t)$$

Where $N^{T}\left(x\right)$ is a row vector containing the shape functions used to approximate the displacement a(t)[40] in the nodes. Using the Galerkin method, we approximate the weighting functions in the same way as the displacement field, producing at the elementary level equation [40] (29):

(30) $$\left(\int_{\Omega^{e}}B\lambda^{n}C_{0}B^{T}d\Omega^{e}\right)a+\int_{\Omega^{e}}Nbd\Omega^{e}+B.C.=0$$

where B is the derivative operator (discrete version of operator $B_{1}$ of equation (6)) that converts the displacements into strains (see [40]). This system of equations is completed by applying the Neumann and Dirichlet conditions suitable for solving the elastic problem (see Figure 1).

Similarly, for the electric case we can use the finite element method. For this purpose we use the weak expression of equation (23) in terms of electrical potential to produce (30):

(31) $$\int_{\Omega }\left(\nabla w\right)\left(\in_{\rho }\lambda^{m}+\delta (f)\right)\left(\nabla \phi \right)d\Omega +\int_{\Omega }w\rho_{e}d\Omega -\int_{\Gamma }w\left(\left(\in_{\rho }\lambda^{m}+\delta (f)\right)\left(\nabla \phi \right)\cdot n\right)d\Gamma =0$$

where w is the weighting function and n is the external normal to the domain of the problem. Again, for calculation of the approximate solution, the scalar field of the electric potential approaches through [40] (31):

(32) $$\phi (x,t)=N^{T}(x)v(t)$$

Where v is the potential at each node of the element, and N(x) are the shape functions for scalar [40] problems. Weighting functions are chosen in the same manner as shape functions, which is through the method of (Bubnov-) Galerkin standard. Therefore, at the elementary level we have the following equation (32):

(33) $$\left[\int_{\Omega }B_{e}\left(\in_{\rho }\lambda^{m}+\delta (f)\right)B_{e}^{T}d\Omega^{e}\right]v+\int_{\Omega }N^{T}\rho_{e}d\Omega +B.C.=0$$

where

(34) $$B_{e}=\left[\begin{array}{cc} \frac{\partial N_{1}}{\partial x} & \frac{\partial N_{1}}{\partial y}\\ \frac{\partial N_{2}}{\partial x} & \frac{\partial N_{2}}{\partial y}\\ \frac{\partial N_{\mathrm{nod}}}{\partial x} & \frac{\partial N_{\mathrm{nod}}}{\partial y} \end{array}\right]$$

where *nod* is the number of nodes of each element.

### Solution to the equation of relative density

This article solves the equation of dimensionless density (or relative) by integrating equation (26) by the method of Euler. For this purpose we define (34):

(35) $$\frac{d\lambda }{dt}=f(x,t_{k},\lambda ,f)=k_{\mathrm{mec}}\left(\lambda^{n-1}\frac{{\bar{U}}_{\mathrm{mec}}}{U_{ref_{m}}}-1\right)+k_{\mathrm{elect}}\frac{\left(\in_{\rho }\lambda^{m}+\delta (f)\right)}{\lambda }\frac{{\bar{U}}_{\mathrm{elect}}}{U_{ref_{e}}}$$

Therefore, the forward Euler method is defined as [41] (35):

(36) $$\lambda_{k+1}=\lambda_{k}+\Delta tf(x,t_{k},\lambda_{k})$$

where $\Delta t=t_{k+1}-t_{k}$ is the integration time interval and k refers to the evaluation of the variable λ at a specific time, i.e.: $\lambda_{k}=\lambda (x,t_{k})$. This method has been used extensively in the prediction of bone density through remodeling [42]. The forward Euler method is of the first order and has the disadvantage of being unstable for large time intervals [41].

Euler's method was implemented in FORTRAN and was coupled with the elastic and electrical problems. For implementation we used an approach based on element (with an elemental average) [39,43].

### Computational model

**Figure 2:**
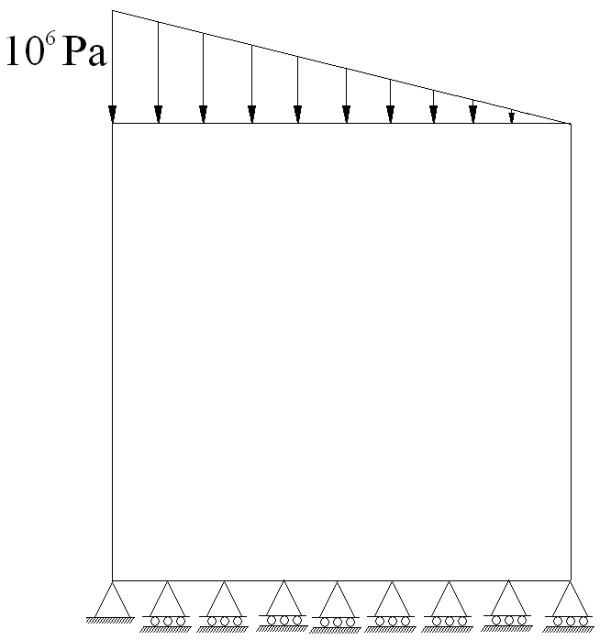
Boundary conditions relating to bone remodeling.

**Figure 3:**
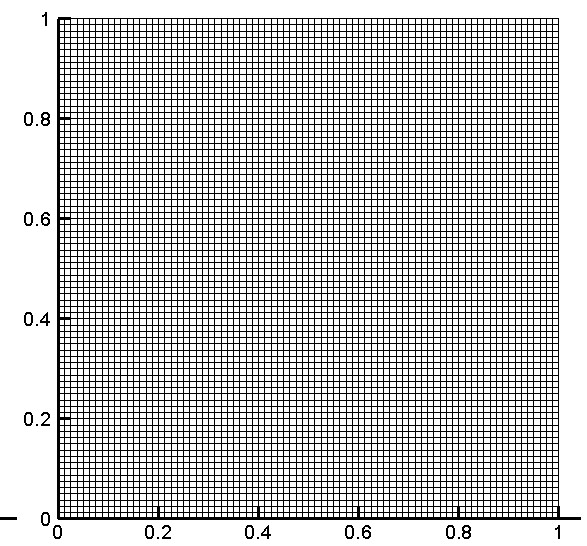
**Mesh used in the example.** (**a**) 80x80.

The computational example to be solved is a 0.1x0.1 m square plate with non-uniform load at the top and restrictions of movement vertically at the bottom as presented in Figure 2. This example has been extensively studied in numerical tests concerning bone remodeling algorithms [42-44]. For these values we built a mesh of 80x80 elements (see Figure 3) in the horizontal and vertical directions, respectively, and integrated these with a time step of Δt=0.100. The total simulation time was 200. The initial condition was λ=1.0 and the limits used in the algorithm of bone remodeling were: 0.0125≤λ≤2.175.

### The constants for the mechanical energy

For the mechanical case we used an initial Young’s modulus $E_{0}$, a Poisson's modulus ν and an initial dimensionless density $\lambda_{0}$ with values 64 MPa, 0.3 and 1.00, respectively [44]. The dimensionless parameters of the density equation were obtained from Nackenhorst (1997) [39,44] and were n=2, $k_{\mathrm{mec}}=0.3125days^{-1}$ and $U_{ref_{m}}=800Pa$.

The mesh was produced using bilinear quadrilateral elements and four points of Gauss integration [40].

### The constants for electric energy

**Figure 4:**
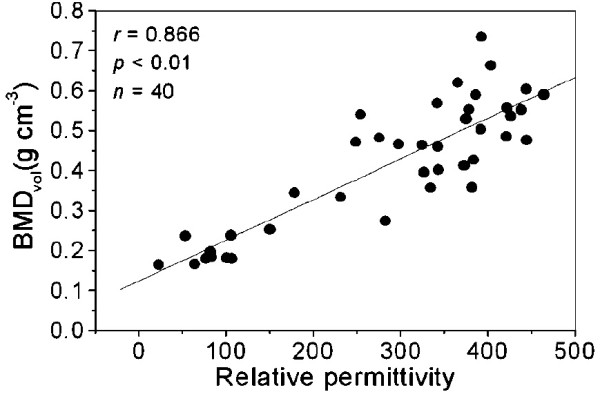
**Bone density as a function of the relative permittivity at a frequency of 50 KHz.** Taken from [46].

**Figure 5:**
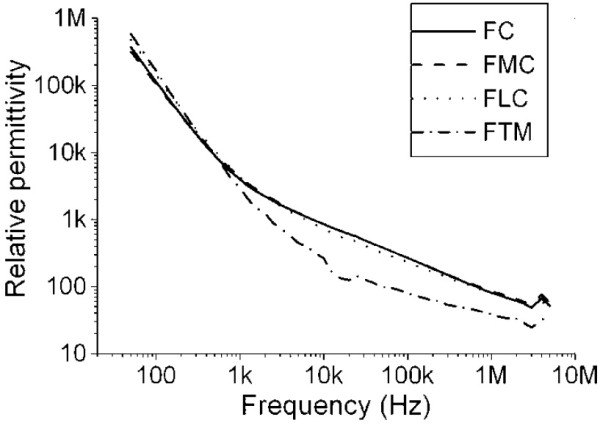
**Graphic representation of relative permittivity (dimensionless) as a function of frequency (in Hz) for various densities of bone tissue.** FC: Femoral head; FMC: femoral medial condyle, FLC: femoral lateral condyle FTM: femoral greater trochanter. Taken from [46].

In the electric case, there are numerous articles [45-48] that determine the major electrical properties of bone. In particular, graphs of the electric permittivity in function of frequency and density are provided in [46], as presented in Figures 4 and 5. Figure 4 presents data concerning bone density as a function of the relative permittivity on the frequency of 50 KHz. The article [46] used 40 bone tissue samples, and used the measurement of the density as a function of the permittivity. From the data, the mathematical model proposed for permittivity as a function of tissue density is given by a power equation, expressed as (36):

(37) $$\in_{\left(\rho \right)}(\rho ,50KHz)=B\rho^{m}$$

Where m=1.5486 and B=1050.0; this is a function of the density of bone tissue. With the aim of using dimensionless density, we multiplied and divided by $\left(\rho_{0}^{n}/\rho_{0}^{n}\right)$ to produce (37):

(38) $$\in_{\left(\rho \right)}(\rho )=1050.0\rho_{0}^{1.5486}\lambda^{1.5486}=\in_{\rho }\lambda^{1.5486}$$

Where $\in_{\rho }=1050.0\rho_{0}^{1.5486}$ and depends on the initial density that considers the computational model.

Figure 5 presents the relative permittivity in terms of the function of the frequency for the density of the femoral head of $\rho =0.3737g.cm^{-3}$. With a greater frequency, permittivity decreases. To complement equation (37) (33) we must add a term that allows us to calculate the difference between the relative permittivity at a frequency of 50 kHz and any other frequencies used in the model; this is (38):

(39) $$\begin{array}{l} \delta_{\left(f\right)}(f)=\in_{r(f)}\left(0.3737g.cm^{-3},f\right)-\in_{r(50Khz)}\left(0.3737g.cm^{-3},f=50Khz\right)\\ \delta_{\left(f\right)}(f)=\in_{r(f)}\left(0.3737g.cm^{-3},f\right)-228.681 \end{array}$$

where $\in_{r(50Khz)}\left(0.3737g.cm^{-3},f=50Khz\right)=228.681$. For its part, the function $\in_{r(f)}\left(0.3737g.cm^{-3},f\right)$ can be obtained from Figure 5, so we get:

(40) $$\log_{10}\left[\in_{r(f)}\left(0.3737g.cm^{-3},f\right)\right]=0.1407{\left[\log_{10}(f)\right]}^{2}-1.8936\log_{10}(f)+8.1505$$

**Figure 6:**
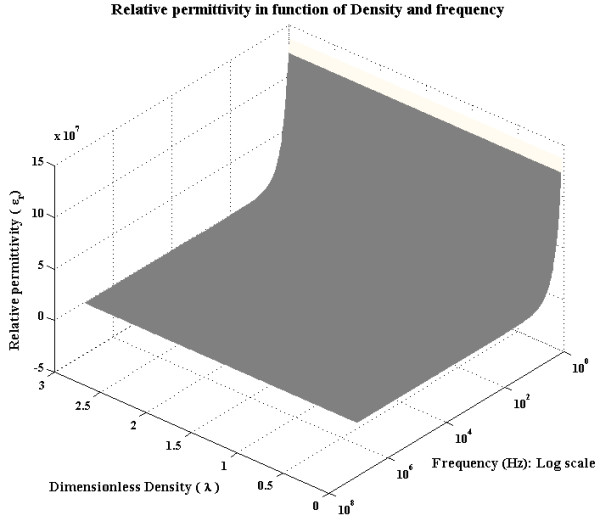
Graphic reconstruction of relative permittivity as a function of frequency and density.

In summary, the equation for the electric permittivity depending on the density and frequency is given by (see Figure 6):

(41) $$\in_{r}(\rho ,f)=\in_{\rho }\lambda^{1.5486}+\in_{r(f)}-228.681$$

For the remaining constant changes we made variations of $k_{\mathrm{elec}}$ and used $U_{ref_{e}}=800Pa$, as in the mechanical case.

## Results

**Figure 7:**
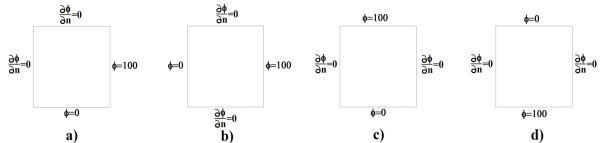
Examples developed in the article; ϕ indicates voltage.

Figure 7 presents various examples that were run for a frequency f = 50 KHz, and with variation in the constant *K*_*elect*_. For these examples, a potential difference at various contour lines was imposed, according to the domain that was established in Figure 2. In the first example, a voltage of 100 was placed on the right side and no voltage at the bottom (Figure 7a). In the second example, a voltage of 100 V was placed on the right side and no voltage on the left (Figure 7b). In the third and fourth examples, the voltage was placed on the top and bottom, respectively (Figures 7c and 7d). In the other contours we placed null Neumann conditions.

**Figure 8:**
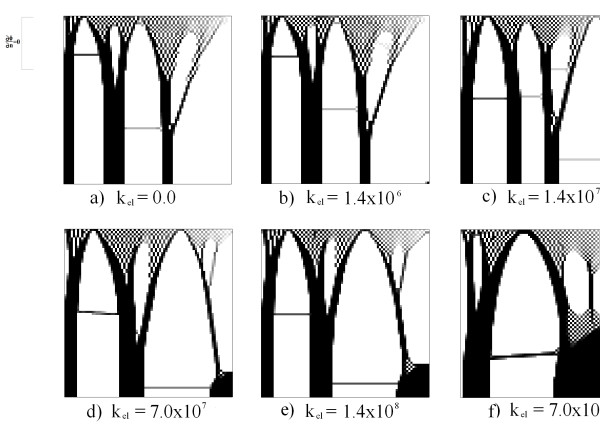
**Results for the first example: the electric boundary condition in Figure**7a. The figure demonstrates the results for various values of k_elect_.

In the first example, the mechanical condition demonstrated in Figure 2 coupled with an electric field as observed in Figure 7a were used. The results are presented in Figure 8.

**Figure 9:**
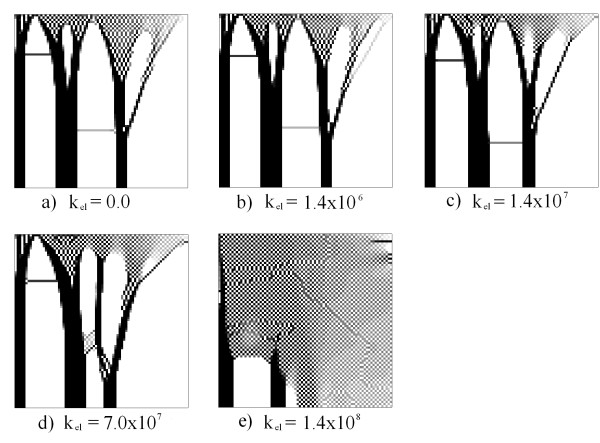
Results for the second example: varying the frequency in equations (35) and (36).

**Figure 10:**
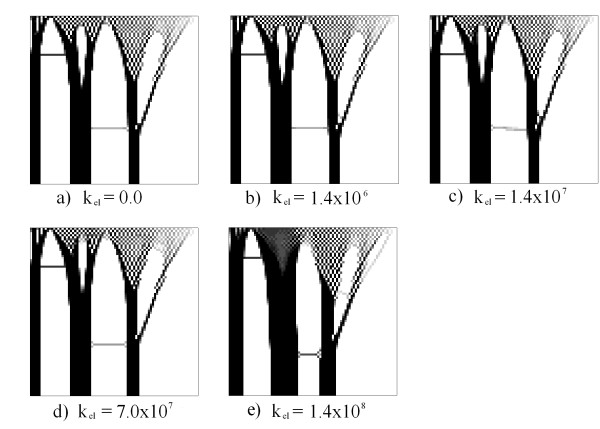
**Results for the first example: the electric boundary condition in Figure**7b. The figure presents results for various values of *k*_*elect*_.

**Figure 11:**
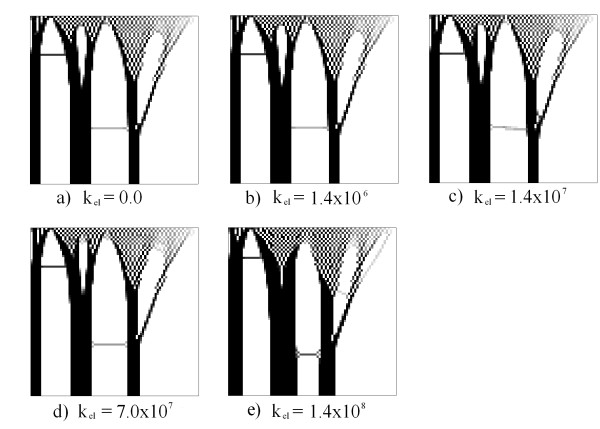
**Results for the first example: the electric boundary condition in Figure**7c. The figure presents results for various values of *k*_*elect*._

**Figure 12:**
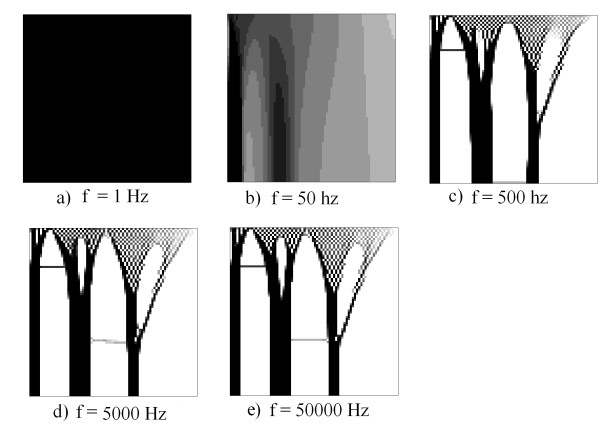
**Results for the first example: the electric boundary condition in Figure**7**d. The figure demonstrates the results for various values of*k***_***elect.***_

Figure 8 presents the results for the relative density value of λ using an approach based on element, with an average at an elementary level of the mechanical stimulus, electrical and density values. In all figures (Figures 8, 9, 10, 11 and 12) black represents the maximum density value (2,175) and white represents the lowest value (0.0125). It should be noted that with Δt = 0.1 the pattern obtained is similar for all values of *k*_*elect*_. It is observed that near the area of imposition of the mechanical stress there is a similar density distribution to a chess board, and far from the loading area there is the formation of three well-defined columns reminiscent of the cortical bone (Figure 8a and 8b). In cases where the constant increases (*k*_*elect*_ = 1.4x10^7^ and above) there is the appearance of a high density area in the lower right region, from where a new column starts in the top domain (Figs 8c, d, e and f). In this area, near the lower right structure, there is the distribution and space formation of a chess board. In addition, in the upper part there are empty areas that generate ramifications in each of the columns supporting the load. Note that the imposition of the electric field defines a new topological structure in the domain, as presented on the right-hand side of the simulation results. This new structure is an additional column, which is generated by the potential and a support area of higher density in the lower right corner.

Figure 9 presents the results for various values of *K*_*elect*_. In this case, a voltage of 100 V was imposed on the right side and a null voltage on the left side. Null Neumann conditions were imposed on remaining contours (see Figure 7b). It is noted that there were changes in topology; columns 2 and 3, from left to right in Figures 9c and 9d, are thicker and closer to one another. In addition, upper branches of greater density were created and an additional branch that begins from the last column to the right. In the case of Figure 9e, the formation of a non-defined region of the "chessboard" type on the right side of the domain is presented.

Figures 10 and 11 present results for various values of *k*_*elect*_. In these cases a voltage of 100 V was imposed at the top and bottom, respectively. Null Neumann conditions were imposed on the remaining contours. Note that in these cases the mass distribution increases as *k*_*elect*_ increases. In the case of Figure 10e, the density increases in the upper part of the domain, so that the chessboard becomes more continuous in the central part of the domain. In Figures 10e and 11e the columns that are formed in each simulation have higher bandwidths than previous cases.

In the second example there is a variation of the frequency, while the value of *k*_*elect*_ remains constant at 7.0x10^7^. With the voltage configuration of Figure 7d, this is with a lower voltage of 100 V. Note in this case that at low frequencies the density and the amount of tissue deposited are greater than at high values. Figure 9a demonstrates that the frequency generates a total deposit of tissue. In Figure 12b the formation of a high density topology and continuing formation is apparent. Figures 12c, 12d and 12e present results comparable to those observed in previous cases. However, in Figure 12c, the columns are wider than in Figures 12d and 12e.

## Discussion and conclusions

In this article several numerical examples were developed concerning bone remodeling of the plate during mechanical stress, assuming the imposition of an electric field in the domain. To calculate the mechanical and electrical stimulus of remodeling, and the evolution of density, the elemental approach was utilized. This pioneering article includes the electrical effect, previously designed by Weinans et al. [4], in a model of bone remodeling.

Comparable with previous articles, the results of the study presented herein demonstrate in the mechanical case formation of the columnar zone (of high density) in the area remote from the load and formation of the trabecular zone (of low density) in the area close to the load [17]. The results are similar to those obtained by Weinans et al. [4], Fernandez et al. [49] and Chen et al. [17]. When applying an electric field there is an increase in bone density and an alteration in the topology of the distribution of mass in the domain. In general, there is greater bone mass apposition in the domain. Therefore, the columns developed by the mechanical stress increase in size due to the electric field. In addition, a greater number of columns and localized compact zones may be observed. In the formulation the effect of electrical frequency has been included to allow increased apposition of mass at low frequency to be observed.

## Competing interests

The authors have no competing interests.

## Authors’ contributions

Work concerning production of the manuscript, modelling and numerical simulation was carried out by each of the authors. All authors read and approved the final manuscript.
